# Lig3-dependent rescue of mouse viability and DNA double-strand break repair by catalytically inactive Lig4

**DOI:** 10.1093/nar/gkae1216

**Published:** 2024-12-14

**Authors:** David Medina-Suárez, Li Han, Sandra O’Reilly, Jiali Liu, Chao Wei, Manon Brenière, Noah J Goff, Chen Chen, Mauro Modesti, Katheryn Meek, Bonnie Harrington, Kefei Yu

**Affiliations:** Department of Microbiology, Genetics and Immunology, Michigan State University, 567 Wilson Rd., East Lansing, MI 48824, USA; Department of Microbiology, Genetics and Immunology, Michigan State University, 567 Wilson Rd., East Lansing, MI 48824, USA; Research Technology Support Facility, and Department of Physiology, Michigan State University, 567 Wilson Rd., East Lansing, MI 48824, USA; Department of Animal Science, Michigan State University, 3018 Interdisciplinary Science and Technology Building, 766 Service Rd, East Lansing, MI 48824, USA; Department of Animal Science, Michigan State University, 3018 Interdisciplinary Science and Technology Building, 766 Service Rd, East Lansing, MI 48824, USA; Cancer Research Center of Marseille, Department of Genome Integrity, CNRS UMR7258, Inserm U1068, Institut Paoli-Calmettes, Aix Marseille Univ, 27 Boulevard Leï Roure CS30059, 13273 Marseille Cedex 09, Marseille, France; Department of Microbiology, Genetics and Immunology, Michigan State University, 567 Wilson Rd., East Lansing, MI 48824, USA; Department of Pathobiology & Diagnostic Investigation, Michigan State University, 567 Wilson Rd., East Lansing, MI 48824, USA; Department of Animal Science, Michigan State University, 3018 Interdisciplinary Science and Technology Building, 766 Service Rd, East Lansing, MI 48824, USA; Cancer Research Center of Marseille, Department of Genome Integrity, CNRS UMR7258, Inserm U1068, Institut Paoli-Calmettes, Aix Marseille Univ, 27 Boulevard Leï Roure CS30059, 13273 Marseille Cedex 09, Marseille, France; Department of Microbiology, Genetics and Immunology, Michigan State University, 567 Wilson Rd., East Lansing, MI 48824, USA; Department of Pathobiology & Diagnostic Investigation, Michigan State University, 567 Wilson Rd., East Lansing, MI 48824, USA; Department of Pathobiology & Diagnostic Investigation, Michigan State University, 567 Wilson Rd., East Lansing, MI 48824, USA; Department of Microbiology, Genetics and Immunology, Michigan State University, 567 Wilson Rd., East Lansing, MI 48824, USA

## Abstract

Recent studies have revealed a structural role for DNA ligase 4 (Lig4) in the maintenance of a repair complex during non-homologous end joining (NHEJ) of DNA double-strand breaks. In cultured cell lines, catalytically inactive Lig4 can partially alleviate the severe DNA repair phenotypes observed in cells lacking Lig4. To study the structural role of Lig4 *in vivo*, a mouse strain harboring a point mutation to Lig4’s catalytic site was generated. In contrast to the ablation of Lig4, catalytically inactive Lig4 mice are born alive. These mice display marked growth retardation and have clear deficits in lymphocyte development. We considered that the milder phenotype results from inactive Lig4 help to recruit another ligase to the repair complex. We next generated a mouse strain deficient for nuclear Lig3. Nuclear Lig3-deficient mice are moderately smaller and have elevated incidences of cerebral ventricle dilation but otherwise appear normal. Strikingly, in experiments crossing these two strains, mice lacking nuclear Lig3 and expressing inactive Lig4 were not obtained. Timed mating revealed that fetuses harboring both mutations underwent resorption, establishing an embryonic lethal genetic interaction. These data suggest that Lig3 is recruited to NHEJ complexes to facilitate end joining in the presence (but not activity) of Lig4.

## Graphical Abstract

**Graphical Abstract:**
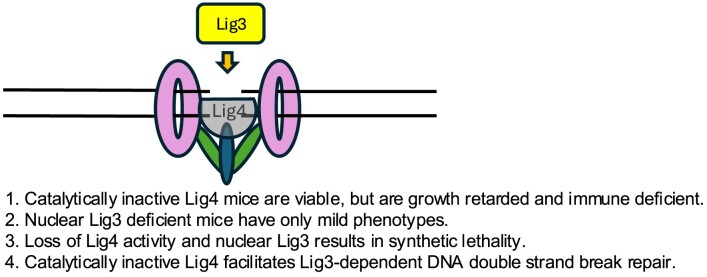

## Introduction

DNA ligases play critical roles in diverse DNA processes including DNA replication, repair and recombination (1,2). The division of labor among the three vertebrate ligases inside the cell has been a point of great interest and debate. The current view is that Lig1 interacts with proliferating cell nuclear antigen (PCNA) and its primary role is ligating Okazaki fragments at replication forks (3–5). Lig1 may also participate in DNA repair such as long-patch excision repair (4). Lig3 interacts with X-ray cross complementing protein 1 (XRCC1) and has been considered as the main ligase for single-strand break (SSB) repair (6–8). DNA ligase 4 (Lig4) interacts tightly with XRCC4 and serves as a dedicated DNA ligase for the major DNA double-strand break (DSB) repair pathway in vertebrate cells, known as non-homologous end joining (NHEJ) (9–11).

Disruption of any of the three DNA ligase genes in mice results in embryonic lethality (12–14). However, cell lines deficient for one or two DNA ligases have been established. Lig4 is not essential for cell line viability, although Lig4-deficient cells typically grow slower and are hypersensitive to ionizing radiation and drugs that induce DSBs (11,15–18). No Lig4 function outside the realm of NHEJ has been reported. Cells deficient for one or more NHEJ factors can still repair DSBs under most circumstances albeit with greatly reduced efficiency, such as immunoglobulin heavy chain gene class switch recombination (CSR), or repair of I-SceI or clustered regularly interspaced short palindromic repeats (CRISPR)-associated protein 9 (Cas9)-induced breaks (15,17,19–21). However, repair of DSBs generated by recombination activation genes (RAGs) during V(D)J recombination absolutely requires core NHEJ factors (e.g. Ku, DNA-PKcs, XRCC4, Lig4, etc.). DSB repair (DSBR) in NHEJ-deficient cells has been collectively termed alternative end joining (A-EJ). A-EJ is likely not a single pathway, and it varies depending on which NHEJ factors are missing (20–23). In the absence of Lig4, either Lig1 or Lig3 must be utilized to join the broken DNA ends, since these are the only remaining DNA ligases in vertebrate cells (17,24).

Lig1 and Lig3 likely evolved from a common ancestor. In vertebrate cells, the mitochondrial DNA ligase is encoded by the Lig3 gene in the nucleus. Some lower eukaryotes (e.g. *Saccharomyces cerevisiae*) do not have Lig3. In those cells, Lig1 encodes the mitochondrial as well as the nuclear DNA ligase (1,2). In vertebrate cells, the mitochondrial DNA ligase is encoded by the *Lig3* gene, which is essential for cell viability. Disruption of the *Lig3* gene, which encodes both a nuclear and a mitochondrial targeted ligase, is only possible if a mitochondrial targeted ligase transgene is preemptively integrated (17,24–27). Surprisingly, nuclear Lig3-deficient cell lines are generally not sensitive to many DNA damaging agents (27,28), suggesting that Lig3 is not essential for nuclear DNA repair. It is likely that nuclear Lig3 function can be mostly replaced by Lig1, establishing that Lig1 and nuclear Lig3 function redundantly. To date, the many efforts to generate cells deficient in both Lig1 and Lig3 have been unsuccessful (16,17,24,25), and the general consensus is that vertebrate cells require either Lig1 or Lig3 for nuclear DNA repair. What has not been addressed is the selective advantage of evolving Lig3 in vertebrates, or the impact of loss of nuclear Lig3 *in vivo*.

Recent studies have discovered a stepwise assembly of NHEJ complexes, transitioning from multiple distinct long-range complexes to a short-range complex (25,29–35). The latter contains Lig4 that plays a structural role in addition to its catalytic activity. Consistent with this notion, yeast and several mammalian cell lines display more efficient DSBR with a catalytically inactive Lig4 over the isogenic lines that completely lack Lig4 (19,36). Moreover, biochemical assays have shown that inactive Lig4 can promote end synapsis and DNA-PKcs autophosphorylation (37), and that intermolecular ligation mediated by Lig3 is enhanced by the addition of inactive Lig4 protein (19). Whether inactive Lig4 facilitates DNA repair *in vivo* has not been addressed. Among the three DNA ligases, Lig3 has a unique zinc finger domain at the N-terminus that greatly enhances its ability to bind DNA breaks and catalyze intermolecular ligation in biochemical assays *in vitro* (38–40). However, a mouse cell line deficient for both Lig3 and Lig4 did not display more severe deficits in DNA repair as compared to cells only deficient in Lig4 (17).

To address the structural role of Lig4 and the function of Lig3 *in vivo*, and the possible interplays among DNA ligases during DSBR, we generated two novel mouse models of DNA ligase deficiency. In one mouse model, a point mutation was introduced into the catalytic site of Lig4 that inactivated Lig4 enzymatic activity. The second mouse model harbors a mutation in the Lig3 gene that disrupts nuclear Lig3 expression while maintaining mitochondrial Lig3. We show that expression of inactive Lig4 in mice has a substantially less severe impact than complete ablation of Lig4, supporting a distinct structural role for Lig4. In contrast to Lig4 ablation or inactivation, loss of nuclear Lig3 has only very minor impacts on mouse physiology underscoring the conundrum of what evolutionary pressure promoted the presence of a third DNA ligase in the nucleus of vertebrate cells. Most interestingly, nuclear Lig3 is clearly essential for the survival of mice harboring inactive Lig4. These results demonstrate substantial cooperativity of DNA ligases 3 and 4 *in vivo* and suggest a potential important benefit for the evolution of a third DNA ligase in the vertebrate nucleus.

## Materials and methods

### Generation and housing of mutant mice

Mutant mice were created by CRISPR-Cas9 genome editing on a C57BL/6 genomic background by MSU’s Transgenic and Genome Editing Facility. Ribonucleoprotein (RNP) complexes were assembled by incubating Cas9 protein and synthetic guide RNA (Integrated DNA Technologies Inc., Coralville, IA). Mouse zygotes were electroporated (Genome Editor GEB15, BEX CO., LTD, Japan) with RNP and single-stranded oligodeoxynucleotide as homology-directed repair templates (HDRT). Electroporated zygotes were implanted into pseudo pregnant recipients using standard embryo transfer procedures. Resultant founder litters were analyzed for targeted edits using Sanger sequencing of purified polymerase chain reaction (PCR) products amplified from genomic DNA extracted from tail biopsies. Mice were maintained in a temperature-controlled environment. Animal husbandry was provided by MSU Campus Animal Resources. Animal protocol (AUF # PROTO202200037) was approved by the Institutional Animal Care and Use Committee at Michigan State University in accordance with the Association for Assessment and Accreditation of Laboratory Animal Care and National Institutes of Health guidelines. Mice were euthanized by carbon dioxide in sealed cages followed by cervical dislocation.

### Lymphocytes preparation and flow cytometry

Single cell suspensions from spleen and thymus were prepared by homogenization followed by passing through a 70 μm cell strainer (VWR, Radnor, PA). Bone Marrow cells were prepared from both femur bones by syringe lavage. Lymphocytes were enriched after lysis of red blood cells in the ACK buffer [0.15 M NH_4_Cl, 10 mM KHCO_3_ and 0.1 mM ethylenediaminetetraacetic acid (EDTA), pH 7.4]. After blocking the Fc receptor with anti-mouse CD16/CD32 (BD Life Sciences, San Jose, CA), cells were stained with fluorescently labeled antibodies (listed below) and analyzed on an Attune CytPix flow cytometer. Flow cytometry data were analyzed using FCS Express v7 (De Novo Software, Pasadena, CA).

### Immunofluorescence and immunohistochemistry

The mouse testes were fixed in 4% paraformaldehyde (P6148, Sigma–Aldrich) overnight at 4°C, and then washed in 0.01 M phosphate-buffered saline (PBS) (pH 7.4), dehydrated in graded ethanol solutions, vitrified with xylene and subsequently embedded in paraffin. Testis sections were cut at a thickness of 5 μm for immunostaining. For immunofluorescence analyses, sections were dewaxed in xylene, rehydrated in graded ethanol solutions and subjected to antigen retrieval by microwaving the sections with sodium citrate buffer (pH 6.0). Following blocking with 5% normal goat serum at room temperature for 30 min, the sections were incubated overnight at 4°C with anti-Lig3 antibody (1:100, 611 876, BD Life Sciences, San Jose, CA) in 5% normal goat serum. Subsequently, after washing with PBS, the sections were incubated with the secondary antibody (1:500, A-21428, Alexa-Fluor 555, Life Technologies) at room temperature in the dark for 1 h. The sections were mounted using Vectorshield mounting media with DAPI (H1200, Vector Laboratory) after three washes with 0.01 M PBS (pH 7.4). Fluorescence was observed and documented using a microscope (EVOS FLc, Life Technologies).

### Macroscopic and microscopic pathology analysis

Following euthanasia, whole brains were fixed in 10% neutral buffered formalin, then sectioned coronally at the caudal aspect of the frontal lobes, mid-thalamus and mid brainstem/cerebellum. The medial to lateral diameter of each of the slit-like lateral ventricles was measured on the caudal aspect of the frontal lobes. Following fixation and macroscopic evaluation, sections of brain were paraffin embedded, sectioned at 4 µm to glass slides and stained with hematoxylin and eosin by routine methodology. Brains were evaluated by a board certified veterinary anatomic pathologist (B.K.H.).

### Plasmid constructs, protein purification and ligation assays.

Plasmid vectors pRSFDuet-1 Lig4 wild type (WT), pRSFDuet-1 Lig4 K273S, pETDuet-1 XRCC4 and pETDuet-1 XLF were generated to express codon optimized mouse Lig4, XRCC4 and XLF in the Rosetta 2 (pLysS) bacterial host strain (Novagen). The Lig4 ORF was engineered to produce a C-terminal poly His tagged polypeptide but the XRCC4 was untagged. Vector pETDuet-1 human XLF has been described (41). The Lig4/XRCC4 complex was produced by co-transformation of cells with the pRSFDuet-1 Lig4 and pETDuet-1 XRCC4 and triple selection in Luria broth (LB) medium containing ampicillin, kanamycin and chloramphenicol. Inductions with isopropyl β-D-1-thiogalactopyranoside (0.5 mM final concentration) were performed at 15°C for 16 h. Expression of XLF (with a cleavable N-terminal poly His tag) was similarly performed but in LB medium containing ampicillin and chloramphenicol. All proteins were purified in a two-step protocol that included Ni-NTA followed by sepharose Q chromatography using procedures described elsewhere (41). All proteins were dialyzed in 150 mM KCl, 20 mM Tris-HCl, pH 7.5, 1 mM EDTA, 1 mM DTT and 10% glycerol, aliquoted, snap frozen in liquid nitrogen and stored at −80°C. Ligation reactions were performed in a 10 μl volume that contained 50 nanograms of XbaI linearized pUC19 DNA, 20 mM Tris-HCl, pH 7.5, 1 mM ATP, 2 mM MgCl_2_, 50 M KCl, 20 nM Lig4/XRCC4 and 200 mM XLF when indicated. Reactions were incubated for 1 h at room temperature, then stopped by addition of 1 μl of proteinase K at 10 mg/ml, 2 μl of 6× sodium dodecyl sulfate (SDS) purple loading dye (NEB) and incubated for 10 min at 55°C before fractionation by electrophoresis on a 0.8% agarose gel in 1x Tris-Borate-EDTA buffer. Gels were stained with ethidium bromide and documented using a ChemiDoc MP Imaging System (Bio-Rad).

#### Isolation of mouse embryonic fibroblasts

Mouse embryos were isolated after timed pregnancy and washed with ice-cold PBS. The head above the eyes and red tissues (heart and liver) were removed. The remaining embryo tissues were homogenized in 0.25% trypsin-EDTA by pulling through a 21G needle with a 1 ml syringe. Homogenates were plated in 10 cm tissue culture dishes containing 10 ml of Dulbecco’s modified Eagle’s medium (high glucose, with L-glutamine) supplemented with 10% fetal bovine serum and (100 U/ml) penicillin–streptomycin. Medium was changed 6 h after plating to remove floating cells and debris. When dishes became confluent, mouse embryonic fibroblasts (MEFs) were harvested and stored (P0), or passage further to set up experiments.

#### Lentivirus

LentiCas9-EGFP was a gift from Phil Sharp & Feng Zhang (Addgene plasmid # 63592). The Cas9 gene in LentiCas9-EGFP was replaced by a mouse nuclear Lig3 gene [no mitochondrial localization sequence (MLS)]. The resulting plasmid was co-transfected with psPAX2 and pMD2.G into HEK293T cells to produce lentiviruses. PsPAX2 and pMD2.G were gifts from Didier Trono (Addgene plasmid # 12260 and 12260). Supernatant harvested at 48 h after transfection was ultracentrifuged over a 20% sucrose cushion for 90 min at 90 000 × *g* to concentrate the virus (by 10-fold). Concentrated viruses were used to infect the MEF with the addition of 4 μg/ml polybrene (Sigma, H9268), resulting in over 90% of the MEFs positive for GFP.

#### Cell fractionation

MEFs or testes were homogenized in ice-cold nuclei isolation buffer [10 mM Hepes, pH 7.9; 15 mM potassium chloride, 2 mM magnesium chloride, 0.1 mM EDTA, 1 mM dithiothreitol and 0.5% nonidet *P*-40 (NP40)] and incubated on ice for 10 min. Nuclei were pelleted by centrifugation for 5 min at 700 × *g* at 4°C. The supernatant was collected as cytoplasmic fraction. The nuclei pellet was homogenized in radioimmunoprecipitation assay (RIPA) buffer [25 mM Tris-HCl, pH 7.5, 150 mM sodium chloride, 1 mM EDTA, 1% NP40, 0.5% sodium deoxycholate and 0.1% SDS). Homogenate was centrifuged for 15 min at 21 000 × *g* at 4°C. The supernatant was collected as nuclear extract. In cases where mitochondria were separated from the cytosol, the cytoplasmic fraction was centrifuged at 25 000 × *g* for 15 min and mitochondria pellet was suspended in RIPA buffer in equal volume to the cytosol.

#### Zeocin sensitivity assay

MEFs were seeded at 10 000 cells per well in 96-well plates. The next day, cells were treated with various amount of zeocin and kept in the incubator for 4 days. After removing the growth medium, 100 μl of 0.5 mg/ml MTT (thiazolyl blue tetrazolium bromide) in PBS was added to each well and incubated for 3 h in the incubator. After the MTT solution was removed, cells were lysed in 250 μl of isopropanol containing 4 mM HCl and 0.1% NP40. The plate was centrifuged for 5 min at 1000 × *g* to pellet the debris, and 200 μl of supernatant from each well was transferred to a new plate for measurement of absorbance at 570 nm.

**Oligonucleotides** (Integrated DNA Technologies, Inc., Coralville, IA)

Protospacer for targeting Lig4: 5′ TACATCGAAACTAAGCTTGA 3′

Lig4 HDRT: 5′ CGCCATCTTTGTGCATCTGCATGCGCTCACCATCAAGacTAGTTTCGATGTAGAAACTCT-GCTGCTTCAT3’. Lig4 genotyping primers: 5′ TGCCAGCAATAACTCTGGCA 3′ and 5′ CGCGCCCTAG-CTTCTTCTTA 3′. Protospacer for targeting Lig3: 5′ GCTGCTCTGCCATCGCACAG 3′. Lig3 HDRT: 5′ TCCTAGCTCTCCAGAGAGGTCATCTAAGACCACGTGCCACCCACCTTACTTTCTGGC-CAGGGTCGCATGTGGGACTCTGTACTGGCCCCTGTGCGAATTCAGAGCAGCGGTTCTGTGTGGACTATGCCAAGCGGGG 3′. Lig3 genotyping primers: 5′ AGCCTGGATTCTTCATGTCTGT 3′ and 5′ TTCGTTA-TCTTCCAGCTCTTCC 3′.

### Antibodies

Rabbit anti-Lig1, Proteintech (18051–1-AP). Mouse anti-Lig3, BD 611876 (raised against a.a. 2–115 of human Lig3). Rabbit anti-Lig4, a gift from David Schatz. Mouse anti-Lamin B1, Proteintech (66095–1-Ig). Mouse anti-GAPDH-HRP, Proteintech (HRP-60004). Mouse anti-β-actin-HRP, Santa Cruz Biotechnology (sc-47778 HRP). Mouse anti-PDH-E1α-HRP, Santa Cruz Biotechnology (sc-377092 HRP). Rat anti-mouse CD16/CD32, BD 553142. Rat anti-mouse IgM, FITC, BD 553437. Rat anti-mouse CD45R/B220, APC, BD 553092. Rat anti-mouse CD19, APC, BD 550992. Rat anti-mouse CD43, FITC, BD 553270. Rat anti-mouse CD3, FITC, BD 561798. Rat anti-mouse CD4, FITC, BD 561828. Rat anti-mouse CD8a, APC, BD 561093.

## Results

### Generation of mouse strain with catalytically inactive Lig4

**Figure 1.**
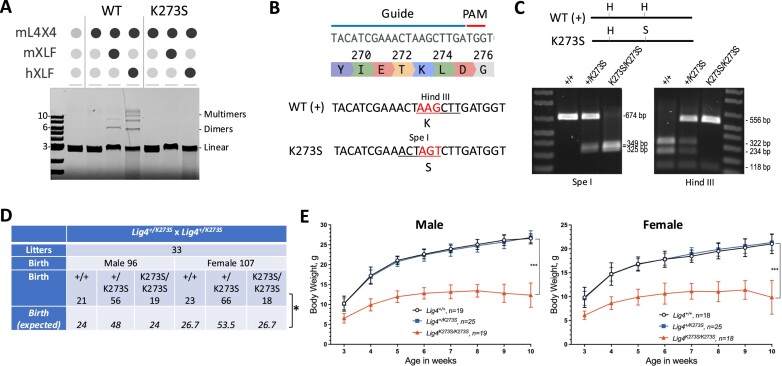
Generation and characterization of mice with inactive Lig4. (**A**) K273S mutation of Lig4 inactivate its enzymatic activity *in vitro* for ligating a linear DNA fragment into dimers and multimers. M, mouse. H, human. X4, XRCC4. L4, Lig4. (**B**) K273S (codon AAG to AGT) mutation was introduced by CRISPR gene editing and homology-directed repair with a single-strand template in fertilized mouse eggs. The guide RNA and PAM sequences are indicated by blue and red lines, respectively. (**C**) Genotyping of mutant allele. PCR amplicon and restriction sites are shown. +, wild type allele. S, Spe I. H, Hind III. (**D**) Mating between *Lig4^+/K273S^* mice yields offspring of all three possible genotypes. *P < 0.05, calculated based on Chi-squared test with Yates continuity correction. (**E**) Both male and female *Lig4^K273S/K273S^* mice are growth retarded as compared to their littermates. WT and *Lig4^+/K273S^* mice have no difference in body weights. Error bars represent standard deviations. ***P< 0.001, based on two-way analysis of variance (ANOVA) with Tukey’s multiple comparisons test.

All DNA ligases have a conserved lysine residue at the catalytic site that becomes adenylated as the first step of the ligation reaction. For mouse Lig4, that lysine is residue 273 (K273). We confirmed the essential role of this lysine in catalysis by showing that recombinant Lig4 protein carrying this mutation fails to ligate DNA fragments *in vitro* (Figure 1A). To study the *in vivo* effect of having a catalytically inactive Lig4, we performed CRISPR genome editing coupled with homology-directed repair (HDR) in fertilized mouse eggs to mutate K273, altering the lysine codon AAG (K) to AGT (S) (Figure 1B). This sequence alteration eliminates a Hind III restriction site while generating a new Spe I site (Figure 1B), facilitating genotyping by PCR followed by restriction enzyme digestions (Figure 1C). One of several heterozygous female founders carrying a WT allele and a K273S allele was chosen to mate with WT C57BL/6 mice to transmit the mutant allele. Repeated crossing to WT C57BL/6 mice were carried out for five generations to dissipate potential CRISPR-associated off-target mutation.

In contrast to the embryonic lethality observed in murine targeted ablation of Lig4 (14), mating between *Lig4^+/K273S^* mice yielded *Lig4^+/+^*, *Lig4^+/K273S^*and *Lig4^K273S/K273S^* offspring, although statistical analyses indicate that the ratios differ modestly from the Mendelian ratios (*P* = 0.0125) (Figure 1D). Male and female mice were born at nearly equal numbers (Figure 1D). The impact of the inactivating Lig4 mutation is clearly less severe than complete ablation of Lig4 (14), substantiating a non-catalytic role for Lig4 *in vivo*, consistent with our previous study using cell lines (19).

The CRISPR-edited embryos also generated founders carrying small indels (e.g. 1 bp insertion or deletion). We propagated a founder carrying the allele with a 1 bp deletion (designated ‘−’) because it causes a frameshift to all downstream coding sequences, resulting in a null allele. The 1 bp deletion was chosen over the 1 bp insertion because it destroyed a Hind III site that can greatly facilitate genotyping by PCR and Hind III digestion. (Supplementary Figure S1A and B). Consistent with previous studies (14), breeding of heterozygous mice yielded no *Lig4^-/-^* pups and we conclude that complete loss of Lig4 results in embryonic lethality (Supplementary Figure S1C).

### Growth retardation and severe lymphocyte development defects of *Lig4^K273S/K273S^* mice

Both male and female *Lig4^K273S/K273S^* mice are severely growth retarded (∼50% the size of littermate controls, Supplementary Figure S2A and Figure 1E), reminiscent of the impact of Ku86 or Ku70 ablation in mice (42,43). *Lig4^K273S/K273S^* mice typically stop growing at the age of 6 weeks (Figure 1E). Between 6–10 weeks of age, *Lig4^K273S/K273S^* mice develop various adverse health issues including corneal opacification and ulceration (37%), malocclusion (30%, Supplementary Figure S2B), weight and fur loss and other complications (33%) that require euthanasia (body condition score < 2, per veterinary guidance). Neither male nor female *Lig4^K273S/K273S^* mice were able to produce offspring. It is unclear whether this reproductive incompetency is due to defects in the reproductive systems or simply because of health complications that mandate euthanasia at relatively young ages.

**Figure 2.**
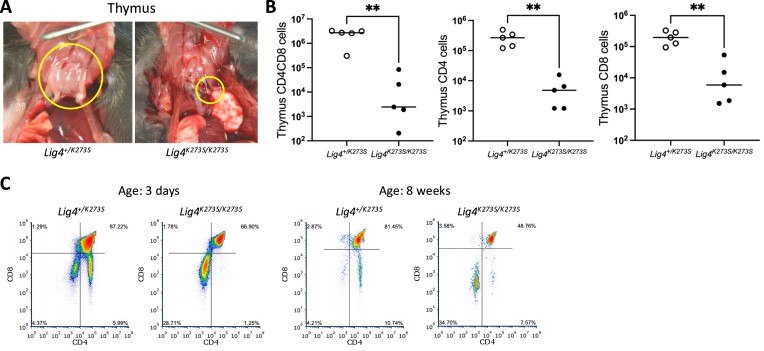
Thymus and thymocyte defects in *Lig4^K273S/K273S^* mice. (**A**) The *Lig4^K273S/K273S^* thymus is tiny and most times barely visible as compared to the *Lig4^+/K273S^* thymus. (**B**) CD4^+^CD8^+^, CD4^+^ and CD8^+^ cells are 100∼1000× fold less in the *Lig4^K273S/K273S^* thymus as compared to the *Lig4^+/K273S^* thymus. (**C**) Flow cytometry analysis of thymic CD4^+^CD8^+^, CD4^+^ and CD8^+^ cells in the *Lig4^K273S/K273S^* thymus at 3 days and 8 weeks after birth. **P< 0.005, based on unpaired t-tests.

**Figure 3.**
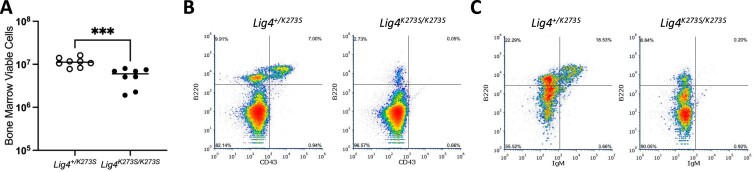
Bone marrow B-cell defect in *Lig4^K273S/K273S^* mice. (**A**) Viable cells isolated from femur bones of *Lig4^+/K273S^* and *Lig4^K273S/K273S^* mice. (**B**) The greatly diminished B lineage cells (B220^+^) in the *Lig4^K273S/K273S^* bone marrow. (**C**) The lack of naïve B cells (IgM^+^) in the *Lig4^K273S/K273S^* bone marrow. ***P < 0.0005, based on unpaired *t*-tests.

**Figure 4.**
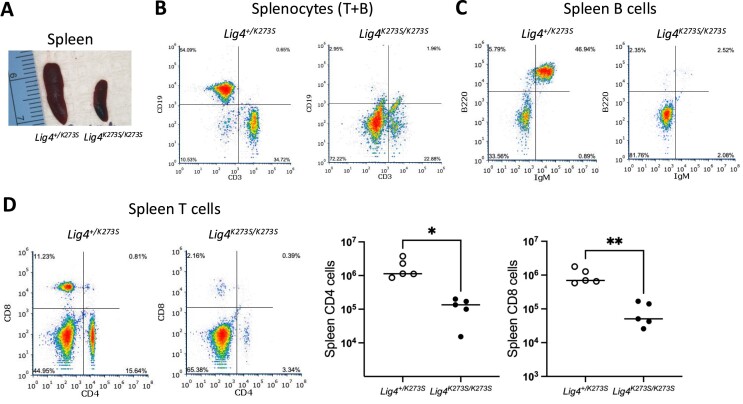
Splenocytes in *Lig4^K273S/K273S^* spleen. (**A**) The *Lig4^K273S/K273S^* spleen is much smaller than that of the *Lig4^+/K273S^* spleen. (**B**) The *Lig4^K273S/K273S^* spleen contains much less T cells (CD3+) and no B cells (CD19+). (**C**) Lack of mature B cell (B220^+^IgM^+^) in the *Lig4^K273S/K273S^* spleen. (**D**) Helper (CD4^+^) and cytotoxic (CD8^+^) T cells are greatly reduced in the *Lig4^K273S/K273S^* spleen. *P < 0.05; **P < 0.005, based on unpaired t-tests.

Like other viable NHEJ deficient mice, *Lig4^K273S/K273S^* mice have severe lymphocyte development defects. The *Lig4^K273S/K273S^* thymus is barely visible (Figure 2A) and contains 100∼1000× fewer thymocytes than their *Lig4^+/K273S^* littermates (Figure 2B). The few recovered thymocytes have a higher percentage of CD4^-^CD8^-^ double negative cells and a lower percentage of CD4^+^CD8^+^ double positive (DP) cells (Figure 2C), suggesting a partial block at the stage of TCRβ rearrangement. This leaky T-cell phenotype resembles that of Ku70-deficient mice (42), but not Ku86-deficient mice as the latter have no DP cells (43). A severe B-cell deficiency was observed in *Lig4^K273S/K273S^* mice as in Ku86 and Ku70 deficient mice. The bone marrow contains slightly less viable cells (Figure 3A), of which very few are B220^+^ cells (Figure 3B) and no IgM^+^ cells (Figure 3C), indicating a severe block of B-cell development at a very early stage. The spleens of *Lig4^K273S/K273S^* mice are significantly smaller than spleens of the *Lig4^+/K273S^* littermates (Figure 4A) and contain ∼10-fold fewer T cells (Figure 4B) and no mature B cells (Figure 4C). Both CD4 and CD8 T cells were detected in the spleen (Figure 4D) but at greatly reduced numbers. The B-cell defect in the spleen is similar to that observed in Ku86 and Ku70 deficient mice.

### Generation of nuclear Lig3 deficient mice

**Figure 5.**
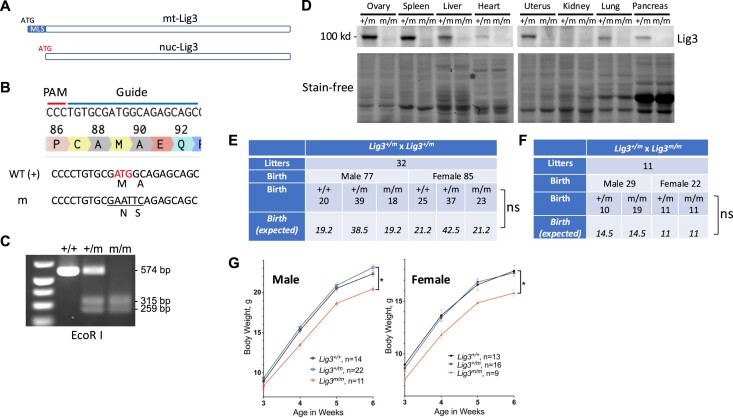
Generation and characterization of mice lack of nuclear Lig3. (**A**) Isoforms of Lig3 peptides resulted from alternative translation initiation target to mitochondrion and nucleus, depending on the presence or absence of a MLS at the N-terminus. (**B**) CRISPR gene editing and HDR in fertilized mouse eggs to generate mutation at the second initiation codon that disable the translation of the nuclear Lig3. The initiation codon for nuclear Lig3 is indicated. The guide RNA and PAM sequences are indicated by blue and red lines, respectively. (**C**) Genotyping of mutant allele by PCR followed by restriction digestion. +, wild type allele. −, mutant allele that disables nuclear Lig3 expression. (**D**) Western blot analysis of Lig3 expression in various tissues in *Lig3^+/m^* and *Lig3^m/m^* mice. Stain-free gel images are shown as loading controls. (**E**) Mating between *Lig3^+/m^* mice results in offspring at near Mendelian ratios. ns, *P* > 0.05. (**F**) Mating between *Lig3^+/m^* and *Lig3^m/m^* mice results in offspring at the expected ratios. ns, *P* > 0.05, calculated based on Chi-squared test with Yates continuity correction. (**G**) Body weights of *Lig3^m/m^* mice as compared to their littermates. Error bars represent standard deviations. *P< 0.05, based on two-way ANOVA with Tukey’s multiple comparisons test.

**Figure 6.**
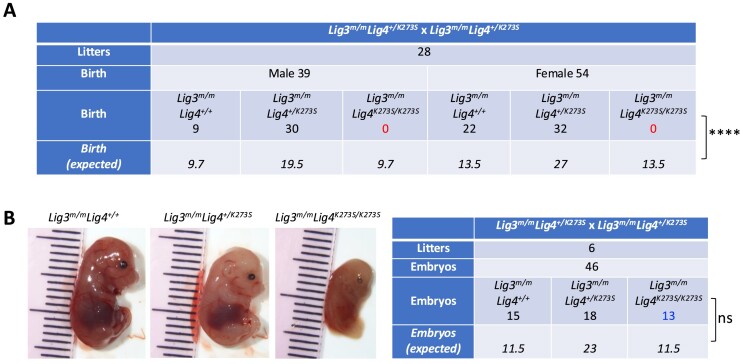
Lig3 is required for the viability of *Lig4^K273S/K273S^* mice. (**A**) Mating between *Lig3^m/m^Lig4^+/K273S^* mice fails to generate *Lig3^m/m^Lig4^K273S/K273S^* offspring. ****P < 0.001, calculated based on Chi-squared test with Yates continuity correction. (**B**) Embryos isolated from pregnant *Lig3^m/m^Lig4^+/K273S^* females at day 15.5 after mating with *Lig3^m/m^Lig4^+/K273S^* male mice. ns, *P* > 0.05, calculated based on Chi-squared test with Yates continuity correction.

Lig3 is expressed in two forms by alternative translation initiation (44) (Figure 5A). Translation from the first ATG generates a peptide containing a MLS that targets the protein to mitochondria (Figure 5A). Alternative translation from the second ATG generates a peptide lacking the MLS and this protein is targeted to the nucleus (Figure 6A). Nuclear Lig3 constitutes the vast majority of cellular Lig3 (17), yet mitochondrial Lig3 is the isoform that is essential for cell viability (27,28). Previously, we designed a strategy that selectively disrupts nuclear Lig3 expression in a mouse B-cell line (17). Here, we employed the same strategy to mutate the Lig3 gene in mouse embryos. CRISPR gene editing coupled with HDR was used to target and mutate the second ATG of the Lig3 gene (Figure 5B). The resulting nucleotide changes generate an EcoR I restriction site (Figure 5B) that allows genotyping via PCR amplification and restriction digestion (Figure 5C). Six founder mice carrying the desired modified allele (hereafter designated ‘m’) were obtained, including one homozygous and five heterozygous mice. One *Lig3^+/m^* founder was selected, and this mouse and its progeny were mated to WT C57BL6 mice for ten generations to dissipate potential CRISPR-induced off-target mutation.

*Lig3* mRNA is expressed ubiquitously at low levels in all tissues except in testis where it is highly expressed (45); western blotting experiments confirmed this expression pattern at the protein level (Supplementary Figure S3A). To confirm that the designed mutation effectively disrupts nuclear expression of Lig3, western blot experiments were performed on lysates of several major organs (Figure 5D). Because the vast majority of Lig3 is in the nucleus, elimination of nuclear Lig3 results in nearly undetectable levels of Lig3 in mutant tissues (Figure 5D). In addition, immunofluorescence staining of the testis (high expression) shows no nuclear staining in *Lig3^m/m^* spermatocytes, round spermatids or sperms (Supplementary Figure S3B). Likewise, no nuclear staining was observed by immunohistochemistry staining of the kidney (low expression) (Supplementary Figure S3C). To determine whether the loss of nuclear Lig3 results in a compensatory overexpression of the other two DNA ligases, western blotting was performed using the lysates of testes of *Lig3^+/m^* and *Lig3^m/m^* mice. As can be seen, Lig1 or Lig4 are not overexpressed in nuclear Lig3 deficient cells (Supplementary Figure S3D). A very low level of Lig3 was detected in the lysate of the *Lig3^m/m^* testis. To determine the source of this low level of Lig3, cell fractionation experiments were carried out to separate the nuclear and cytoplasmic fractions of the testis tissue. A very weak signal was detected in the nuclear fraction of the *Lig3^m/m^* testis extract (Supplementary Figure S3E). Although cell fractionation experiments are often not completely successful in separating nuclear and cytoplasmic components, there is a possibility that *Lig3^m/m^* cells may contain very low levels of nuclear Lig3.

Mating between *Lig3^+/m^* mice resulted in offspring at the expected Mendelian ratios for each genotype (Figure 5E) and approximately equal numbers of males and females. Similarly, mating between *Lig3^+/m^* and *Lig3^m/m^* mice also generated offspring at the expected ratios (Figure 5F). As expected, mating between *Lig3^m/m^* mice generated exclusively *Lig3^m/m^* offspring with an average litter size of five, suggesting no reproductive defect in *Lig3^m/m^* mice. No major differences were observed between WT, *Lig3^+/m^* and *Lig3^m/m^* mice regarding organismal survival. However, a modest impact on birth weight and growth was apparent. Both male and female *Lig3^m/m^* mice weigh significantly less (∼88%) than their littermates (Figure 5F). There is no difference in body weight between the WT and *Lig3^+/m^* mice (Figure 5F). The reason for the reduced body weight in nuclear Lig3 deficient mice is unclear. No tumors were observed in either young (3∼4 weeks) or old (>1 year) *Lig3^m/m^* mice. Examinations of hematoxylin and eosin-stained tissue sections from heart, lung, liver, kidney, spleen and small intestines showed no overt pathology. Therefore, nuclear Lig3 deficiency has only a modest impact on mouse physiology.

### High incidence of cerebral ventricle dilation in*Lig3^m/m^*mice

Although no obvious pathology was observed in most mouse tissues, in the brain, a high incidence of ventricle dilation was observed in the *Lig3^m/m^* mice. Increased ventricular dilation can occur because of increased production of CSF or because of decreased brain volume; *Lig3^m/m^* mice typically have smaller brains as compared to their WT or *Lig3^+/m^* littermates (Supplementary Figure S4A) and display higher incidence of ventricle dilation (Supplementary Figure S4B). Notably, the more severe ventricle dilation (2.0 mm) was only observed in *Lig3^m/m^* mice. Interestingly, whereas ventricle dilation was frequent in relatively old mice (13 and 20 weeks) (Supplementary Figure S4B), in a large cohort of 6-week-old mice, the incidence of ventricle dilation was very low. No ventricle dilation of 2.0 mm was observed in these young mice, and only two *Lig3^+/m^* and two *Lig3^m/m^* mice had a ventricle dilation of 1.0 mm (Supplementary Figure S4C). These data suggest that ventricle dilation develops gradually as the mice age, possibly due to slow accumulation of DNA damage that results in cell loss. As a highly metabolic organ with high oxygen demand, the brain is more likely to accumulate more oxidative damage-related abnormalities than other organs when DNA repair is compromised (46,47). These data imply that Lig3 plays an important role in DNA repair *in vivo*, at least for certain tissues that are more prone to accumulate DNA damages.

### Lig3 is essential for the viability of mice harboring inactive Lig4

In studies assessing end joining in yeast and cultured cells when Lig4 is catalytically inactive, it was speculated that another DNA ligase can catalyze ligation in the context of NHEJ complexes. However, there is no genetic evidence to support this conclusion. Our previous study has shown that nuclear Lig3 deficiency in a *Lig4^-/-^* mouse B-cell line does not further diminish CSR or cellular resistance to zeocin (17). To determine whether nuclear Lig3 is relevant to end joining when Lig4 activity is inhibited *in vivo*, genetic crosses were performed between the two murine models described in this study. Because *Lig4^K273S/K273S^* mice are infertile, *Lig3^m/m^* mice were first crossed with *Lig4^+/K273S^* mice to obtain *Lig3^+/m^Lig4^+/K273S^* mice and subsequently *Lig3^m/m^Lig4^+/K273S^* mice. In crosses between male and female *Lig3^m/m^Lig4^+/K273S^* mice, only *Lig3^m/m^Lig4^+/+^* and *Lig3^m/m^Lig4^+/K273S^* offspring were obtained; no viable *Lig3^m/m^Lig4^K273S/K273S^* pups were born (*P*= 1.855 × 10^–7^) (Figure 6A), suggesting that lack of nuclear Lig3 combined with inactive Lig4 results in embryonic lethality. Timed pregnancies between *Lig3^m/m^Lig4^+/K273S^* mice yielded Mendelian ratios of *Lig3^m/m^Lig4^K273S/K273S^*day 15.5 embryos (Figure 6B); however, the *Lig3^m/m^Lig4^K273S/K273S^* embryos were clearly undergoing fetal resorption (Figure 6B), indicating an embryonic lethality phenotype. These data demonstrate that *in vivo*, Lig3 can function to promote end joining when Lig4 catalysis is blocked, but not when Lig4 is completely absent; these data further implicate the important structural role of Lig4 during end joining.

### DNA end joining in ligase deficient MEFs

To determine the interplay between these two DNA ligases, MEFs of various genotypes were isolated. MEFs of all genotypes except for *Lig3^m/m^Lig4^K273S/K273S^* were readily isolated from day 13.5 embryos. However, day 13.5 *Lig3^m/m^Lig4^K273S/K273S^* embryos are highly deformed and fragile and contain almost no viable cells. Thus, we attempted to isolate MEFs of this genotype from day 11.5 embryos; we were able to isolate a limited number of *Lig3^m/m^Lig4^K273S/K273S^* MEFs; these cells have an extreme proliferation deficit consistent with a dramatically more severe cellular defect compared to cells with either mutation alone.

**Figure 7.**
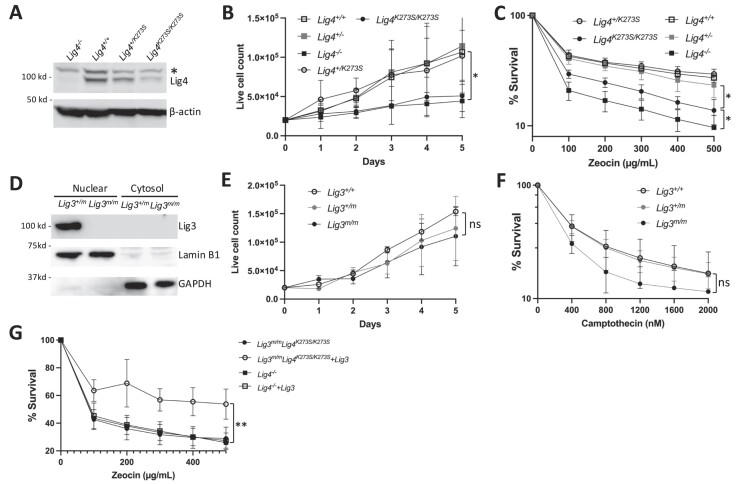
DNA repair in MEFs. (**A**) Western blot analysis of Lig4 expression in MEFs of various genotypes. Asterisk indicates a non-specific band that is cross-reactive to the Lig4 antibody. (**B**) Growth curves of MEFs of various genotypes. (**C**) MTT assays of zeocin sensitivity of MEFs of various genotypes. (**D**) Cell fractionation and western blot analysis of Lig3 in MEFs of the indicated genotypes. (**E**) Growth curves of MEFs of the indicated genotypes. (**F**) MTT assays of camptothecin sensitivity of MEFs of the indicated genotypes. (**G**) MTT assays of zeocin sensitivity of *Lig4^-/-^* and *Lig3^m/m^Lig4^K273S/K273S^* MEFs transduced with lentivirus with a mouse nuclear Lig3 complementary DNA (cDNA). Error bars indicate standard deviations from at least three independent experiments. *P < 0.05; **P< 0.005; ns, *P* > 0.05, based on unpaired *t*-tests.

To document cellular differences between each genotype, cell proliferation assays were performed. As expected, *Lig4^-/-^* MEFs proliferate much slower than *Lig4^+/+^* or *Lig4^+/-^* MEFs (Figure 7B). MEFs in this study proliferate more poorly than the equivalent genotypes described in a previous study (14); this is likely due to mouse strain differences (i.e. C57BL/6 versus 129/SV × C57BL/6). *Lig4^K273S/K273S^* MEFs proliferate similarly to *Lig4^-/-^* MEFs, while *Lig4^+/K273S^* MEFs proliferate similar to WT MEFs (Figure 7B). As expected, *Lig4^-/-^* MEFs are highly sensitive to DSB-inducing drug zeocin as compared to *Lig4^+/+^* and *Lig4^+/-^* MEFs (Figure 7C). Although *Lig4^K273S/K273S^* MEFs are also more sensitive to zeocin than cells with WT alleles, the *Lig4^K273S/K273S^* MEFs are more radioresistant than *Lig4^-/-^* MEFs (Figure 7C). *Lig4^+/K273S^* MEFs showed zeocin sensitivity similar to WT (Figure 7C). Altogether, these data demonstrate that a catalytic inactive Lig4 can partially rescue DSB repair over having no Lig4 at all. In cell culture models, catalytically inactive Lig4 supports substantial levels of episomal end joining (19). We posit that this difference may be because this murine K273S allele is relatively poorly expressed compared to WT Lig4 (Figure 7A).

Lig3 nuclear deficiency alone does not affect the proliferation of MEFs (Figure 7E), or their sensitivity to DSB-inducing drugs (data not shown). Consistent with our previous study with cell lines (17), nuclear Lig3 deficiency leads to increased sensitivity to a DNA topoisomerase I inhibitor, camptothecin (Figure 7F), although the difference did not reach statistical significance.

To determine whether Lig4 truly plays a structural role in helping Lig3 to participate in DSB repair, a nuclear Lig3 encoding cDNA was transduced into *Lig4^K273S/K273S^* and *Lig4^-/-^* MEFs by lentivirus. Although *Lig4^-/-^* MEFs are healthier than *Lig4^K273S/K273S^* MEFs, zeocin resistance was partially restored only in Lig3-transduced *Lig4^K273S/K273S^* MEFs, but not in Lig3-transduced *Lig4^-/-^* MEFs (Figure 7G). These data are consistent with our previous cell line study (19) and further strengthen our conclusion that inactive Lig4 significantly facilitates Lig3 mediated end joining, providing direct evidence for a structural role for Lig4 in DSB repair.

## Discussions

In this study, two DNA ligase deficient mouse models were generated. In one model, the catalytic site of Lig4 was mutated to disable the enzymatic activity while preserving the protein’s structural integrity allowing an *in vivo* assessment of a previously proposed structural role for Lig4. Mice harboring inactive Lig4 are viable, in contrast to the embryonic lethality observed when Lig4 is totally ablated. The fact that a catalytically inactive Lig4 is sufficient for DNA repair both *in vitro* and *in vivo* strongly argues for a critical non-catalytic, most likely structural, role of Lig4 in DSBR.

This finding leads to two important points. First, it suggests that the DNA ends in the recently reported NHEJ short-range complex are accessible to other DNA ligases or other DNA modifying enzymes. The existing short-range complex structures leave little or no space for insertion of a second ligase or modifying enzymes (34,48). This suggests that there is more flexibility in the synapsis models than can be appreciated in the long-range/short-range scheme. The flexible synapsis model is one alternative for which there is both cellular (*in vivo*) and sm-FRET support (35,49,50). Second, from a genetic standpoint, the inactive Lig4 and the backup DNA ligase collaborate instead of competing to join the DNA breaks. It is not clear how DNA ends in the short-range complex are transferred to the heterologous ligase and whether the heterologous ligase needs to entirely replace the inactive Lig4 in the assembled short-range complex. In other words, is this collaboration a temporal transfer or a physical collaboration?

In comparison to several other NHEJ deficient mouse models, the growth and lymphocyte development defects in mice with inactive Lig4 are on par with those observed in Ku86 and Ku70 deficient mice (42,43,51), but are milder than those observed in *XRCC4* or *Lig4-null* mice (embryonic lethal) (14,52), and are more severe than those observed in *scid* or *DNA-PKcs-null* mice (53–56). Like the Ku86 and Ku70 deficient mice, B-cell deficiency is more prominent than that of T cells, even though both cell types require V(D)J recombination (which is dependent on NHEJ) to rearrange their antigen receptor genes. The reason for the differential impact of NHEJ deficiency on B and T cells is unclear but may reflect different tolerances of B and T cells to DNA damage.

The other mouse model in this study is nuclear Lig3 deficiency. Although Lig3 exists in all vertebrates and some lower eukaryotic organisms, its function in nuclear DNA repair has been enigmatic. The general view of Lig3 as the primary ligase for single strand break repair is inferred from its interaction with XRCC1, which is a critical scaffold protein in this repair pathway. However, several cell lines engineered to be devoid of nuclear Lig3 are mostly insensitive to DNA damaging agents, raising the question of Lig3’s role in nuclear DNA repair. The likely explanation, supported by several different studies, is that Lig1 can efficiently perform most, if not all, of the nuclear DNA repair normally carried out by Lig3. Our study here is the first to examine this apparent functional redundancy *in vivo*. We utilized a strategy previously used in our cell culture model to disrupt nuclear Lig3 expression by a point mutation that abrogates the nuclear-specific translation start site in the protein. Consistent with a minimal impact on DNA repair observed in Lig3-deficient cell lines *in vitro*, nuclear Lig3 deficiency has a minimal impact on mouse physiology *in vivo*. Other than a modest reduction of body weight and a higher incidence of cerebral ventricle dilation in old mice, no overt pathology has been observed in mice deficient in nuclear Lig3. Because Lig3 affects cellular abundance of XRCC1, it is also possible that the mild phenotypes are associated with a mild reduction of XRCC1.

The most interesting finding in this study is that nuclear Lig3 is essential for the viability of mice with an inactive Lig4. This provides direct evidence that Lig3 plays an essential role as a backup ligase in DSBR that was apparent in the limited cell line studies when Lig4 is completely ablated (17,24,25). Although a possible role for Lig1 as a backup for Lig4 cannot be excluded, this study demonstrates that Lig1 cannot fully replace Lig3 *in vivo*. Another implication is that although Lig3 is robust for intermolecular ligation of DSBs in biochemical assays, its full capacity for DSBR *in vivo* requires the physical presence of Lig4. Despite the genetic evidence provided here, the exact molecular mechanism by which Lig3 is recruited to the Lig4-dependent NHEJ complex remains unsolved. A more interesting question is whether Lig3 mediates some end-joining events in NHEJ proficient cells. This is a difficult question because there is no signature that allows unequivocal identification of Lig3-mediated joining. Although increased junctional microhomology (MH) usages at the joints are often used to implicate deviations from classical NHEJ, it is worth pointing out that classical NHEJ also strongly favors a few base pairs of MH, and that a slight increase of MH is not a reliable measure to distinguish alternative end joining from classical NHEJ (57). Loss of function mutations of NHEJ factors are very detrimental to human cells and human patients with NHEJ defects are very rare. In human patients that harbor hypomorphic Lig4 mutations, it is conceivable that some DSBR might be mediated by Lig3. Whether the potential shift of ligase usage has any pathological effect would be a point of interest for future investigations.

## Supplementary Material

gkae1216_Supplemental_File

## Data Availability

The authors confirm that the data supporting the findings of this study are available within the article and its supplementary materials.

## Notes

Present address: Jiali Liu, State Key Laboratory of Animal Biotech Breeding, College of Biological Sciences, China Agricultural University, Beijing, 100193, China.

Present address: Bonnie Harrington, Charles River Laboratories International, Inc., Mattawan, MI, 49071, USA.
