# Supplementary material for: Lig3-dependent rescue of mouse viability and DNA double-strand break repair by catalytically inactive Lig4
Source: Nucleic Acids Res. 2024 Dec 14;53(2):gkae1216. doi: 10.1093/nar/gkae1216 (PMC11754673; doi:10.1093/nar/gkae1216)
Supplement: gkae1216_Supplemental_File [file gkae1216_supplemental_file.pdf]

Supplementary Figure 1. A. The null allele (-) with a 1 bp deletion was generated by CRISPR/Cas9-mediated gene editing in fertilized mouse eggs. The guide RNA and PAM sequences are indicated by blue and red lines, respectively. **B.** Genotyping of null allele. PCR amplicon and restriction sites are shown. +, wild type allele. -, null allele with 1 bp deletion. H, Hind III. **C.** Mating between *Lig4*$^{+/-}$ mice results in embryonic lethality of *Lig4*$^{-/-}$ offspring. *, $p < 0.05$, calculated based on Chi-Squared test with Yates continuity correction.

Supplementary Figure 2. Physical appearances of *Lig4*$^{K273S/K273S}$ mice. A. *Lig4*$^{K273S/K273S}$ mice are about half the size of the *Lig4*$^{+/K273S}$ littermates. **B.** Frequent malocclusion in *Lig4*$^{K273S/K273S}$ mice.

Supplementary Figure 3. A. Lig3 protein expression level in various mouse tissues. Homogenate from 1 mg of tissue was loaded to each lane and probed with anti-Lig3 antibody by western blotting. **B.** Immunofluorescence staining of sections of testis for nuclear Lig3 expression. **C.** Immunohistochemistry staining of sections of kidneys for nuclear Lig3 expression. **D.** Western blot analysis of the three DNA ligases in the testes of *Lig3*$^{+/m}$ and *Lig3*$^{m/m}$ mice. **E.** Cell fractionation and western blot analysis of testis tissue from *Lig3*$^{+/m}$ and *Lig3*$^{m/m}$ mouse, respectively. The XRCC1 signals in cytoplasmic and mitochondrial fractions may potentially come from nuclei contamination during the cell fractionation.

Supplementary Figure 4. A. Sections of fixed mouse brains showing the ventricle dilations. Solid lines indicate 1.0mm and dashed lines indicate 2.0mm. **B.** Cases of cerebral ventricle dilations in mice of different genotypes at ages 13 and 20 weeks, respectively. **C.** Cases of cerebral ventricle dilations in mice of different genotypes at the age of 6 weeks.

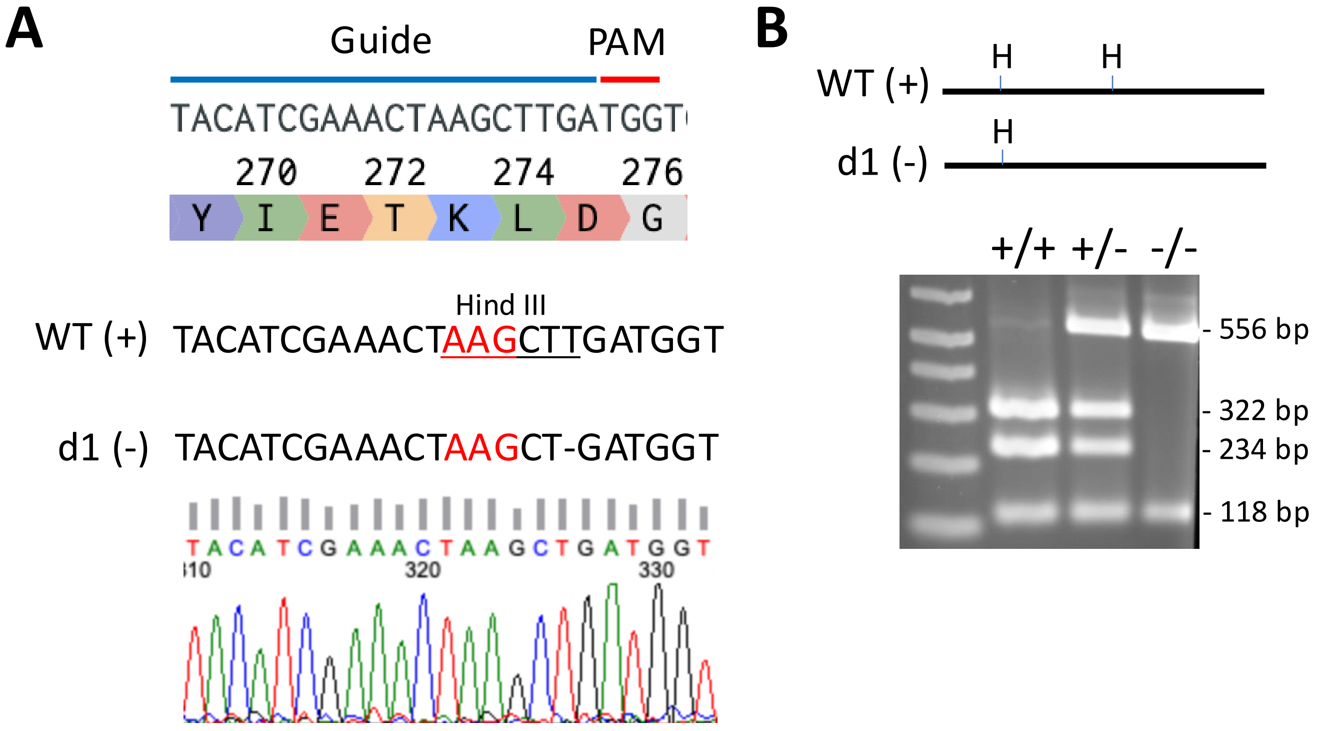

C

	Lig4+/- x Lig4+/-					
**Litters**	4					
**Birth**	Male 19			Female 7		
**Birth**	+/+<br>6	+/-<br>13	-/-<br>0	+/+<br>1	+/-<br>6	-/-<br>0
Birth (expected)	*4.8*	*9.5*	*4.8*	*1.8*	*3.5*	*1.8*

*

Figure S1

A

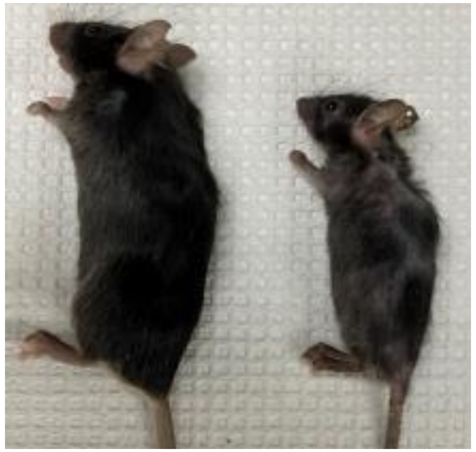

B

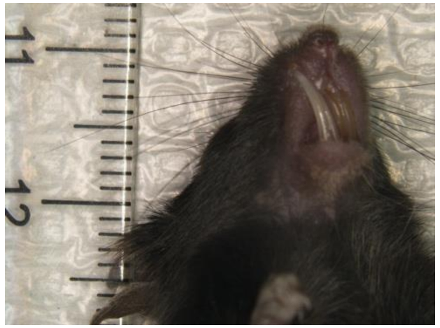

Figure S2

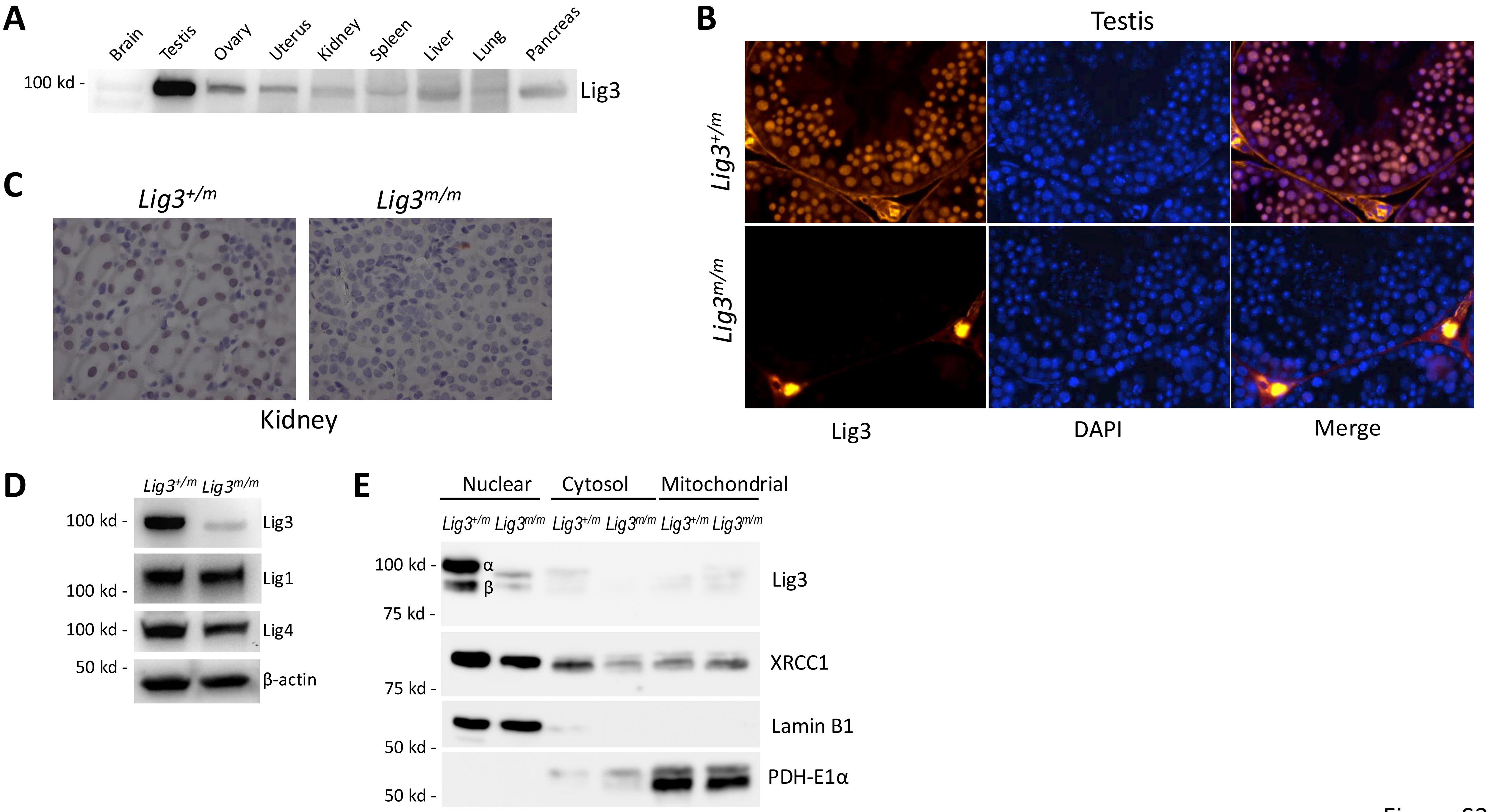
A
Brain
Testis
Ovary
Uterus
Kidney
Spleen
Liver
Lung
Pancreas
100 kd -
Lig3
B
Testis
Lig3+/m
Lig3m/m
Lig3
DAPI
Merge
C
Lig3+/m
Lig3m/m
Kidney
D
Lig3+/m
Lig3m/m
100 kd -
Lig3
Lig1
100 kd -
100 kd -
Lig4
50 kd -
β-actin
E
Nuclear
Cytosol
Mitochondrial
Lig3+/m
Lig3m/m
Lig3+/m
Lig3m/m
Lig3+/m
Lig3m/m
100 kd -
α
β
75 kd -
Lig3
XRCC1
75 kd -
Lamin B1
50 kd -
PDH-E1α
50 kd -

Figure S3

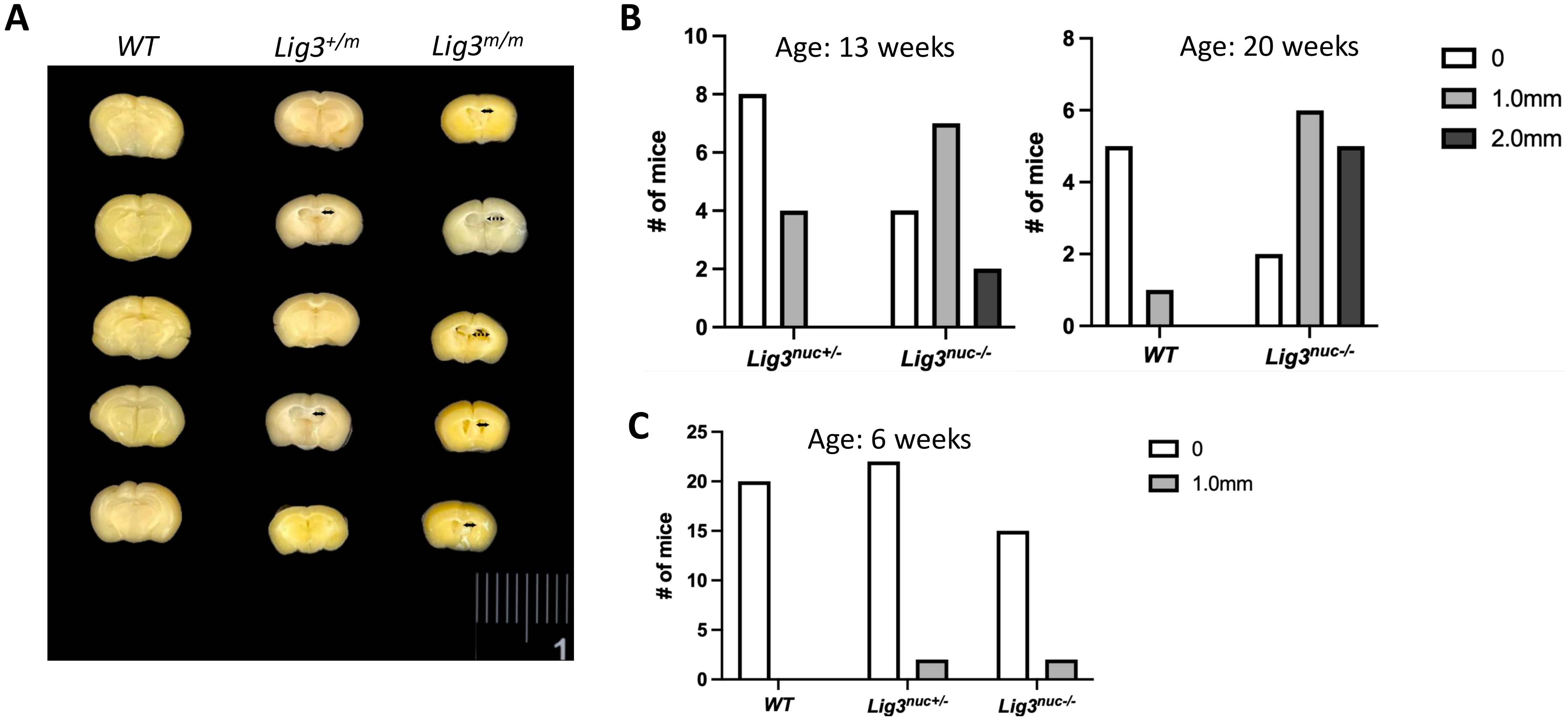
A
WT
Lig3+/m
Lig3m/m
1
B
Age: 13 weeks
# of mice
10
8
6
4
2
0
Lig3nuc+/-
Lig3nuc-/-
Age: 20 weeks
# of mice
8
6
4
2
0
WT
Lig3nuc-/-
0
1.0mm
2.0mm
C
Age: 6 weeks
# of mice
25
20
15
10
5
0
WT
Lig3nuc+/-
Lig3nuc-/-
0
1.0mm

Figure S4
